# Tripartite-motif family genes associated with cancer stem cells affect tumor progression and can assist in the clinical prognosis of kidney renal clear cell carcinoma

**DOI:** 10.7150/ijms.51260

**Published:** 2020-10-18

**Authors:** Guangzhen Wu, Yingkun Xu, Lin Li, Jianyi Li, Ningke Ruan, Jian Dong, Zhuyuan Si, Qinghua Xia, Qifei Wang

**Affiliations:** 1 Department of Urology, The First Affiliated Hospital of Dalian Medical University, Dalian, Liaoning, 116011, China; 2 Department of Urology, Shandong Provincial Hospital, Cheeloo College of Medicine, Shandong University, Jinan, Shandong, 250021, China; 3 Department of Orthopedics, Shandong Provincial Hospital, Cheeloo College of Medicine, Shandong University, Jinan, Shandong, 250021, China; 4 The Nursing College of Zhengzhou University, Zhengzhou, Henan, 450001, China; 5 Department of Sports Medicine and Adult Reconstructive Surgery, The Affiliated Drum Tower Hospital of Nanjing University School of Medicine, Nanjing, Jiangsu, 210008, China; 6 Department of Hepatobiliary Surgery, Shandong Provincial Hospital, Cheeloo College of Medicine, Shandong University, Jinan, Shandong, 250021, China; 7 Department of Urology, Shandong Provincial Hospital Affiliated to Shandong First Medical University, Jinan, Shandong, 250021, China

**Keywords:** TRIM, kidney renal clear cell carcinoma, TCGA, cancer stem cells, survival

## Abstract

Ubiquitination is presently a hot topic in the field of oncology. The tripartite-motif (TRIM) family of proteins represents one of the largest classes of putative single protein RING-finger E3 ubiquitin ligases, which play an essential role in the ubiquitination of proteins in the body. At the same time, research related to cancer stem cells (CSCs) is increasing in popularity in the field of oncology. CSCs are potentially chemically resistant and can be selectively enriched in patients receiving chemotherapy, ultimately leading to adverse outcomes, such as treatment failure and cancer recurrence. There is a close relationship between multiple TRIM family genes and CSCs. Accumulating evidence suggests that TRIM family proteins are expressed in diverse human cancers and act as regulators of oncoproteins or tumor suppressor proteins. In this study, we used biological information to explore the potential function of TRIM family genes related to CSCs in the development of pan-cancer. Kidney renal clear cell carcinoma (KIRC) is one of the deadliest malignant tumors in the world. Owing to its complex molecular and cellular heterogeneity, the effectiveness of existing KIRC-related risk prediction models is not satisfactory at present. Therefore, we focused on the potential role of these TRIM family genes in KIRC and used seven TRIM family genes to establish a prognostic risk model. This model includes TRIM16, TRIM32, TRIM24, TRIM8, TRIM27, PML, and TRIM11. In conclusion, this study provides further insight into the prognosis of KIRC, which may guide treatment.

## Introduction

Ubiquitination is achieved through an enzymatic cascade in which ubiquitin is activated through a covalent bond with an E1 ubiquitin-activating enzyme. This activation depends on ATP-driven adenylation of the protein, which then covalently binds the protein to the E1 enzyme via a thioester bond. It then transfers the activated protein to the E2 protein-binding enzyme via trans-thioesterification^1^. In the final step, E3 ubiquitin ligase mediates the transfer of ubiquitin to lysine residues in the substrate^2^. The covalently linked ubiquitin then acts as a receptor for another ubiquitin molecule, eventually producing a long ubiquitin chain^3^. The key regulatory steps of the ubiquitination reaction are determined by E3 ubiquitin ligase, which determines the choice of substrates and selection of polyubiquitin chains^4^. Changes in the Notch, Wnt, and TGF-β signaling pathways have been confirmed to exist in most cancers^5^. These pathways play a significant role in regulating cell proliferation^6^. Particularly in the case of cancer, the relationship between Notch, Wnt, and TGF-β signaling pathways and ubiquitination is very close^7^.

The tripartite-motif (TRIM) protein family has more than 80 family members in humans. They are involved in many different cellular processes but also have RING-mediated E3 ubiquitin ligase activity functional characteristics for different proteins^8^. TRIM proteins are a large family of E3 ubiquitin ligases. They all have an N-terminal TRIM consisting of a RING domain, one or two B-boxes, and a coiled-coil domain^9^. The TRIM protein family regulates cell proliferation^10^ and the cell cycle^11^, affects EMT and cell metabolism^12^, and regulates autophagy^13^ and epigenetic modifications^14^. At present, studies are increasingly emphasizing the relationship between TRIM family members and cell stemness. Increasing evidence suggests that several TRIM family genes allow cancer cells to acquire stem cell properties and maintain stem cell-like phenotypes using different mechanisms^15^. For example, in breast cancer cells, TRIM25 promotes metastasis by increasing the stemness and tumorigenicity of cancer cells, thereby enhancing its ability to colonize and grow at the secondary site^16^.

We obtained 13 TRIM family genes (TRIM6, TRIM8, TRIM11, TRIM14, TRIM16, PML, TRIM21, TRIM24, TRIM25, TRIM27, TRIM28, TRIM32, and TRIM71) associated with tumor stem cells from a study by Jaworska et al. 15. In this study, we analyzed the mRNA expression, mutations, and prognosis of these TRIM family genes in pan-cancer. Subsequently, we found that most of these genes have statistical significance in the prognosis of patients with KIRC. Due to our previous research involving KIRC 17, we focused on the potential role of these genes, particularly their mRNA expression, in KIRC and displayed this information in the form of heat maps and violin diagrams. We used the online tool of the GSCALite website to map the TRIM family genes and cancer pathway regulatory network. The GDSC database was used to explore the sensitivity between these TRIM family genes and anticancer drugs. In particular, we used the LASSO regression curve to establish a model related to the prognosis of patients with KIRC, including seven genes; TRIM16, TRIM32, TRIM24, TRIM8, TRIM27, PML, and TRIM11. Based on this model, the AUC of the five-year ROC curve was found to be 0.685, and the AUC of the seven-year ROC curve was found to be 0.707, indicating that this model has an excellent predictive performance. Finally, we mapped the interaction network between target genes and related transcription factors. In short, we believe that our research could provide valuable information for future clinical diagnosis and scientific research.

## Materials and Methods

### Data collection

TCGA website (https://cancergenome.nih.gov/) contains comprehensive cancer genome data, including mutations, copy number variations (CNVs), mRNA expression, miRNA expression, and methylation data. In February 2020, gene mRNA expression data, CNV data, SNV data, and clinical parameters related to patients with KIRC were downloaded from TCGA website. The KIRC dataset in TCGA database includes 72 normal samples and 539 renal clear-cell carcinoma samples.

### GSCALite website

GSCALite (http://bioinfo.life.hust.edu.cn/web/GSCALite/) is an interactive web application based on the TCGA database for genomic cancer analysis that analyzes and visualizes genome expression/variation/correlation in cancer flexibly^18^. The analysis provided by GSCALite includes gene differential expression, overall survival, single nucleotide variation (SNV), CNV, methylation, pathway activity, miRNA regulation, normal tissue expression, and drug sensitivity. The data on this website were used to draw a regulatory network between these TRIM family genes and cancer pathways. The GDSC database was used to explore the sensitivity between these TRIM family genes and anticancer drugs.

### GEPIA website

GEPIA (http://gepia.cancer-pku.cn/) is based on TCGA database and was used to analyze RNA sequence expression data of more than 9,000 tumors and 8,000 tumor genome maps^19^. The website's online tools were used to explore the relationship between these 13 TRIM family genes and the prognosis of various cancers. P < 0.05 was considered statistically significant.

### STRING website

The STRING database (https://string‐db.org/) is an online search tool for analyzing gene or protein interactions. It contains a proven and predicted biological database of protein-protein interactions^20^. To conduct GO and KEGG analyses, this website was used to identify genes related to these 13 TRIM family genes. The results of the GO analysis are displayed in the form of a bubble chart. The results of KEGG analysis are presented as a histogram.

### 2.5. KM-Express website

KM-Express (http://ec2-52-201-246-161.compute1.amazonaws.com/kmexpress/index.php) is an online tool for survival and gene expression analyses of patient samples and cell lines, providing researchers with survival analysis, cross-datasets, and subgroup expression comparisons, as well as expression comparisons between cell lines. Experimental support was provided to identify potential biomarkers for downstream functional studies 21. This website was used to analyze seven TRIM target family genes in various renal cancer cell lines.

### Kaplan-Meier Plotter website

Kaplan-Meier Plotter (https://kmplot.com/analysis/) is an online analysis website based on the TCGA database to explore the prognostic factors of malignant tumors. It can evaluate the influence of up to 54,000 gene expressions on the prognosis of 21 cancer types^22^. We used the Kaplan-Meier Plotter website to analyze the prognostic significance of seven target gene expression in KIRC.

### Cistrome website

Cistrome (http://cistrome.org/) assists with building comprehensive analysis pipelines and performing effective data integration to better uncover hidden biological insights from publicly available high-throughput data. It can use TCGA expression profiles and public ChIP-seq profiles to predict transcription factor targets in cancer. In this study, this website was used to analyze the correlation between the seven TRIM target genes and transcription factors and draw a network of interactions between the target genes and transcription factors.

### Bioinformatics analysis and statistical analysis

Perl language is the acronym for practical extraction and report language. It was initially designed and written by Larry Wall in 1987. This language is an excellent programming language that built on the advantages of many other programming languages. To obtain clinical information more suitable for our research from TCGA database, we first used the Perl language to process these raw data. The R language became available around 1980 and is an outstanding statistical calculation and mapping software widely used in the field of statistics. The bio-information and statistical analyses designed in this study are mainly conducted through various program packages on multiple R language platforms. The Limma package was applied to the analysis chip and RNA-Seq difference analysis. The heat map in the study was drawn using the Pheatmap software package. The Ggplot2 package was used to draw a violin diagram showing the expression of 13 TRIM family genes in KIRC. TCGA KIRC dataset contained biological information for 539 tumor and 72 normal tissues. Co-expression analysis between the two molecules was performed using the Corrplot software package. Subsequently, the Consensus Cluster Plus package was used for cluster analysis. Univariate Cox regression analysis was performed on the 13 TRIM family genes in KIRC. Then, the Glmnet and Survival packages were used for Lasso regression analysis. The genes of seven TRIM families were used to establish a risk signature related to the prognosis of patients with KIRC. The Survival package was used to analyze and draw the survival curve. Then, the Survival ROC software package was used to generate the five- and seven-year ROC curves to verify the accuracy of the model. Finally, according to this risk model, univariate and multivariate Cox regression analyses were performed. In particular, we mapped the expression of transcription factors in patients with KIRC and mapped the regulatory network between target genes and related transcription factors. To improve the credibility of the results, the data in the KIRC dataset of TCGA database were used to conduct random internal sampling validation. Within this dataset containing 539 patients with KIRC, we randomly selected data from 218 of them. Subsequently, based on the risk model, we performed survival curve analysis, ROC curve analysis, univariate Cox regression analysis, and multivariate Cox regression analysis. The above series of results prove once again that our conclusion is reliable. P < 0.05 was considered statistically significant.

## Results

### The landscape of 13 TRIM family genes in various tumors

**Figure 1:**
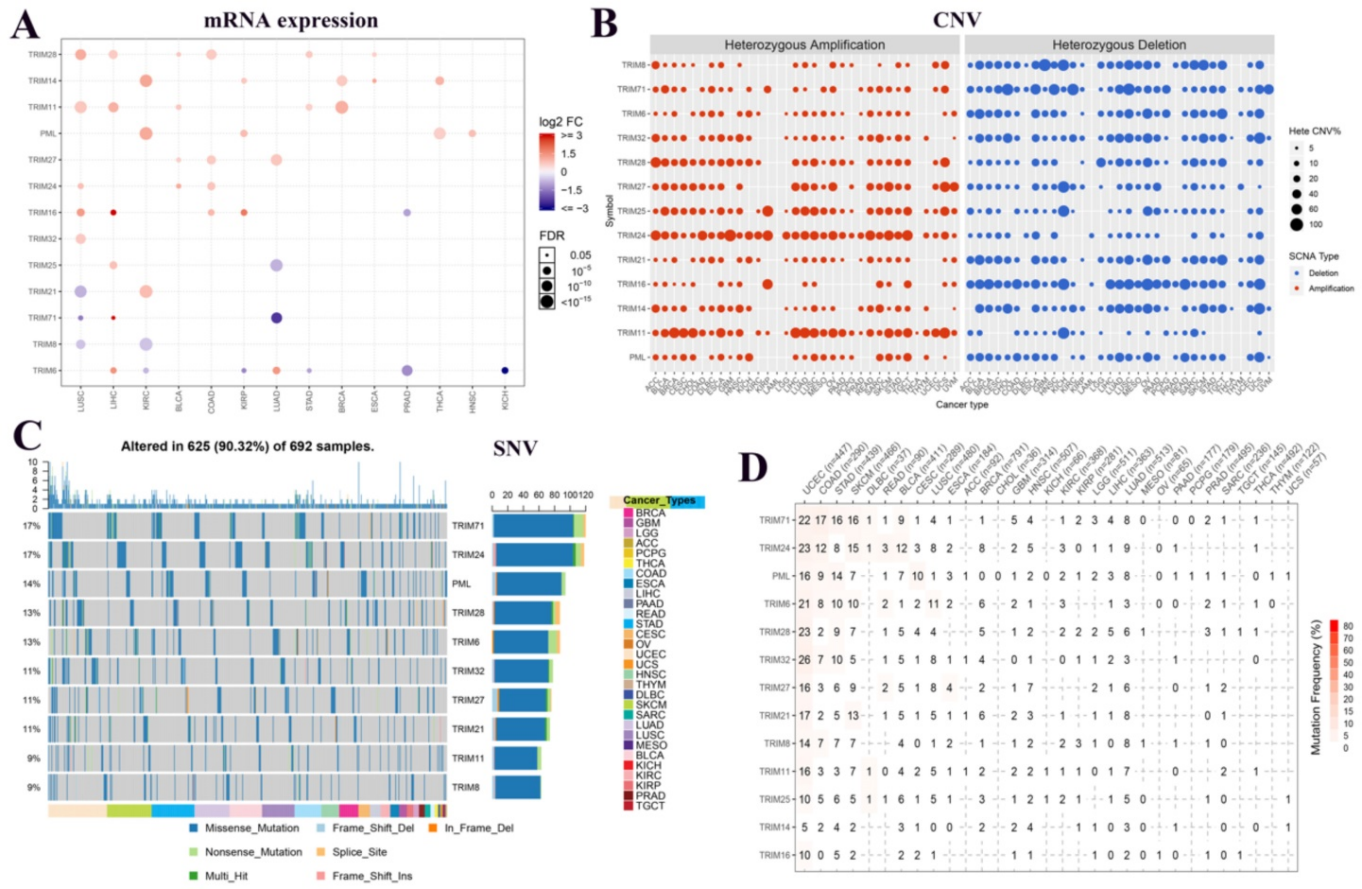
Expression and variation of TRIM family genes related to cancer stem cells in various tumors. (A) The expression of these 13 genes in multiple tumors. Red indicates high expression and blue indicates low expression. The larger the circle, the more statistically significant. (B) The copy number variation (CNV) of the genes in various tumors. In the CNV images, red represents the enlarged mutation, blue represents the missing mutation, and the larger the circle, the higher the mutation rate. (C) Single nucleotide variants of the top 10 TRIM family genes in various tumors. (D) Quantitative table of single nucleotide variations of the genes in multiple tumors.

To understand the expression changes and mutations of these 13 TRIM family genes in multiple tumors, we plotted the mRNA expression, CNV, and SNV of these genes in various tumors. In the panorama of mRNA expression, we observed that TRIM14, PML, and TRIM21 were highly expressed in KIRC. TRIM25 and TRIM17 were expressed at low levels in LUAD (Fig. 1A). In CNV, we observed that TRIM24 had more gain mutations and TRIM16 has more loss mutations in pan-cancer (Fig. 1B). In SNVs, TRIM71 and TRIM24 were present in up to 17% of all tumors. PML, TRIM28, TRIM6, TRIM32, TRIM27, and TRIM21 also had more than 10% SNVs in all tumors. Only the top 10 TRIM family genes are shown in Figure 1C. All information regarding the SNVs is quantified as images in Figure 1D.

### Co-expression analysis of the 13 TRIM family genes and the relationship between expression in pan-cancer and overall survival

**Figure 2:**
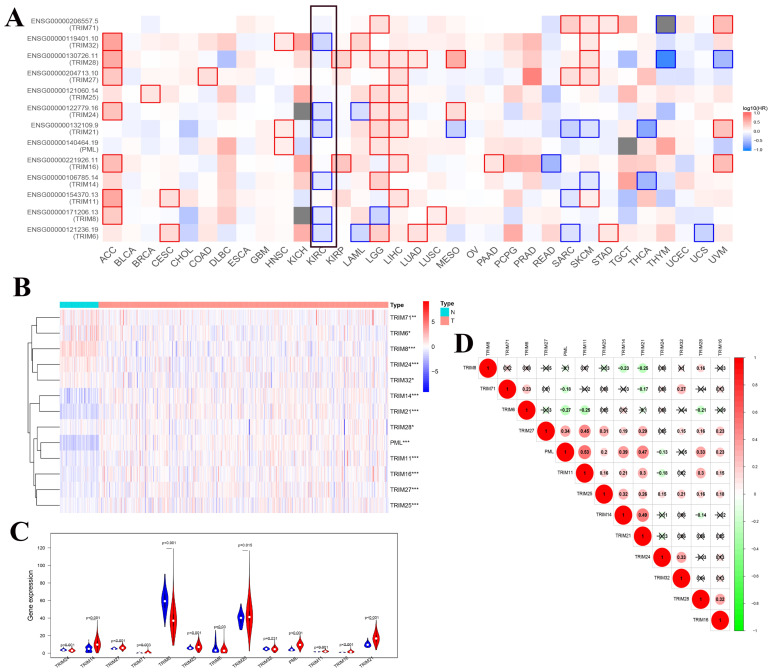
Co-expression analysis of the 13 TRIM family genes and the relationship between expression in pan-cancer and overall survival. (A) Exploration of the overall survival of these 13 TRIM family genes in various tumors through the GEPIA website. The black frame indicates overall survival related to KIRC. (B) The expression levels of the genes in KIRC from TCGA. The higher the expression, the deeper the red color; the lower the expression, the deeper the blue color. (C) The expression of the genes in KIRC. Blue represents normal tissue and red represents tumor tissue. (D) Co-expression analysis of the genes. Red represents a positive correlation and green represents a negative correlation. *P < 0.05, **P < 0.01, and ***P < 0.001.

To determine whether these genes were related to overall survival in a variety of tumors, we used GEPIA's online tool to generate a map of the relationship between these 13 TRIM family genes and the overall survival of multiple tumors. We found that multiple TRIM family genes (TRIM32, TRIM24, TRIM21, TRIM14, TRIM8, and TRIM6) had statistical significance on the overall survival rate of patients with KIRC (Fig. 2A). The heat maps of these 13 TRIM families in the KIRC of TCGA were generated using R software. Among them, 72 were normal and 539 were tumors (Fig. 2B). The expression levels of TRIM14, PML, and TRIM21 were significantly higher in kidney cancer tissues than in normal tissues. The expression levels of TRIM8 were substantially lower in kidney cancer tissues than in normal tissues. The subsequent violin plots further visualized the results in the heatmap (Fig. 2C). Finally, we explored the correlation between the 13 TRIM family genes. The relationship between TRIM11 and PML was the strongest (Fig. 2D).

### GO, KEGG, and GDSC analyses and a regulatory map of genes and related biological pathways

**Figure 3:**
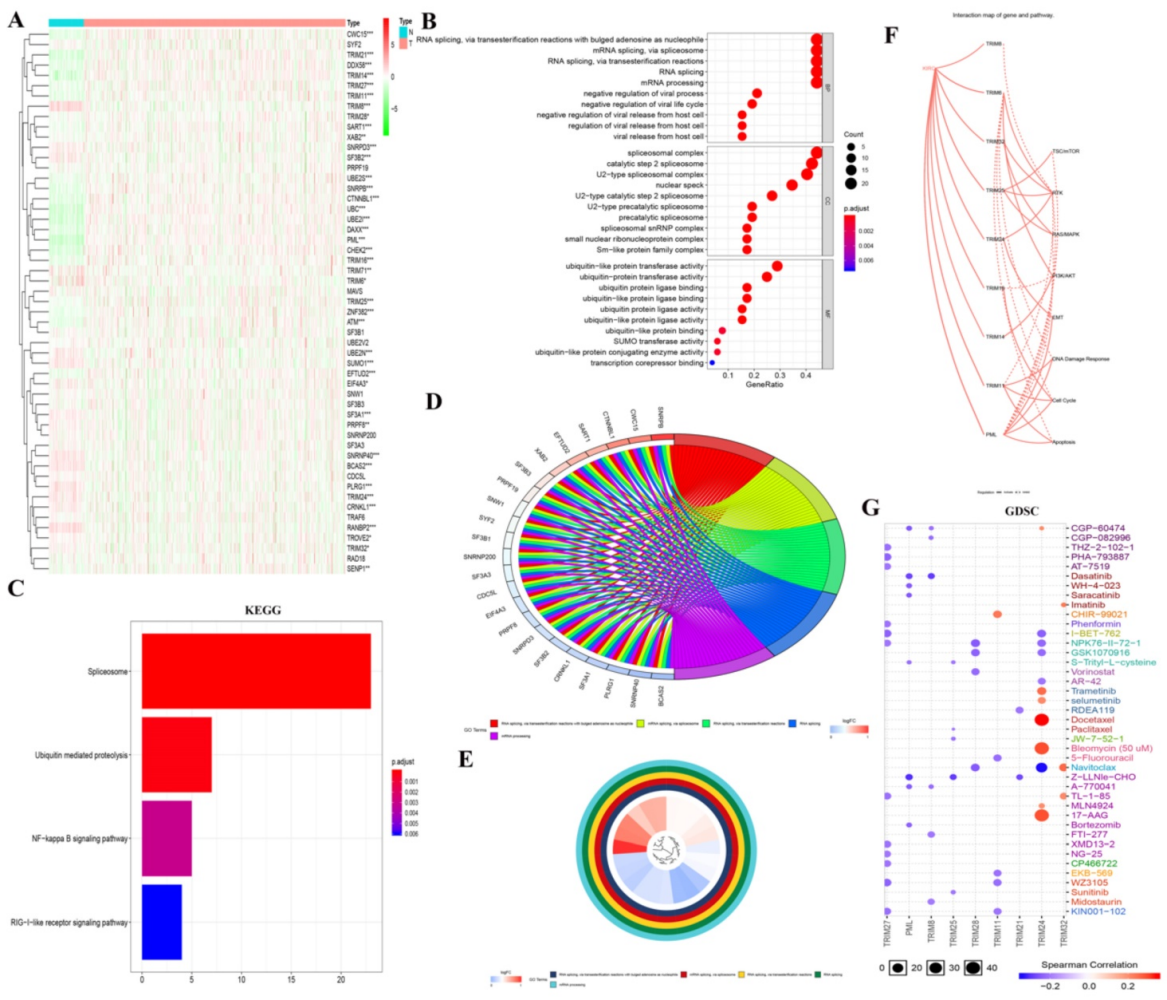
GO, KEGG, and GDSC analyses and a regulatory map of genes and related biological pathways. (A) The heat maps of the genes related to the 13 TRIM family genes were generated by R language in the KIRC database of TCGA, where red indicates high expression and green indicates low expression. (B-E) GO and KEGG analyses of genes related to the 13 TRIM family genes. (F) Analysis of the interactions between these genes and related pathways with the GSCALite online analysis tool. A solid line indicates promotion and a dashed line indicates inhibition. (G) GDSC analysis of the genes related to the 13 TRIM family genes. The color represents Spearman's correlation. The size represents the strength of drug targeting. The larger the circle size, the better the drug sensitivity. *P < 0.05, **P < 0.01, and ***P < 0.001.

To determine how these genes produced biological effects in KIRC, we obtained 13 TRIM family-related genes through the STRING website. We then generated a heatmap of these genes in the KIRC of TCGA using R software (Fig. 3A). Then, GO analysis was performed, where i: RNA splicing via transesterification reactions with bulged adenosine as a nucleophile; ii: mRNA splicing via spliceosome; and iii: RNA splicing via transesterification reactions were displayed in BP; i: spliceosomal complex; ii: catalytic step 2 spliceosome; and iii: U2-type spliceosomal complex were shown in CC; and i: ubiquitin-like protein transferase activity; ii: ubiquitin-protein transferase activity; and iii: ubiquitin protein ligase binding were displayed in MF (Fig. 3B). In particular, the results of GO (BP) analysis are shown in Fig. 3D and 3E. The results of the KEGG analysis showed i: the spliceosome, ii: ubiquitin-mediated proteolysis, and iii: the NF-κB signaling pathway (Fig. 3C). In the interaction network of genes and biological pathways, TRIM24 and PML can promote EMT (Fig. 3F). In GDSC analysis, TRIM24 was strongly sensitive to docetaxel, bleomycin (50 uM), and 17-AAG (Fig. 3G).

### Cluster analysis and correlation with clinical features

**Figure 4:**
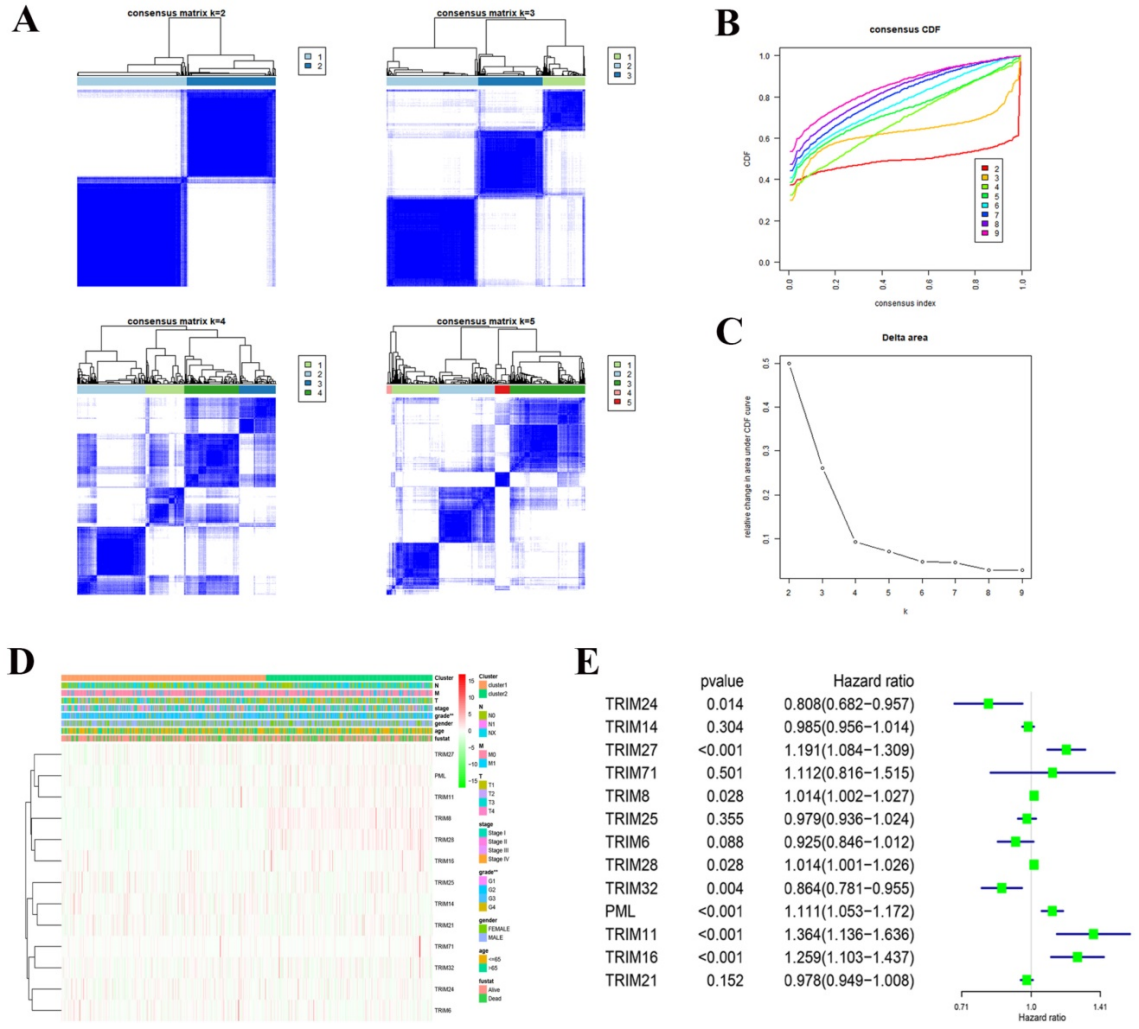
Cluster analysis and correlation with clinical features. (A) Consensus clustering matrix fork = 2-5. (B) Consensus clustering cumulative distribution function (CDF) fork = 2-9. (C) Relative change in area under the CDF curve fork = 2-9. (D) Heatmap and clinicopathologic features of the two clusters (cluster1/2) defined by the 13 TRIM family genes consensus expression. A deeper shade of red indicates higher expression and a deeper shade of green indicates lower expression. (E) Univariate Cox regression calculated the hazard ratio of the 13 TRIM family genes with a 95% confidence interval. *P < 0.05, **P < 0.01, and ***P < 0.001.

Consensus clustering of 13 TRIM family genes identified two KIRC clusters and verified the reliability of the cluster analysis results (Fig. 4A-C). We also explored the relationship with clinical characteristics. We found that the prognosis and grade of patients with KIRC were statistically significant (Fig. 4D). After performing univariate Cox regression analysis in KIRC, we found that the P values of TRIM24, TRIM27, TRIM8, TRIM28, TRIM32, PML, TRIM11, and TRIM16 were less than 0.05, which was statistically significant for prognosis. Among them, the hazard ratio of TRIM24 and TRIM32 was less than 1, playing a protective role in KIRC, and the hazard ratio of TRIM27, TRIM8, TRIM28, PML, TRIM11, and TRIM16 was greater than 1, which plays the role of risk factor in KIRC (Fig. 4E).

### The prognostic value of risk signature and its relationship with clinical characteristics

**Figure 5:**
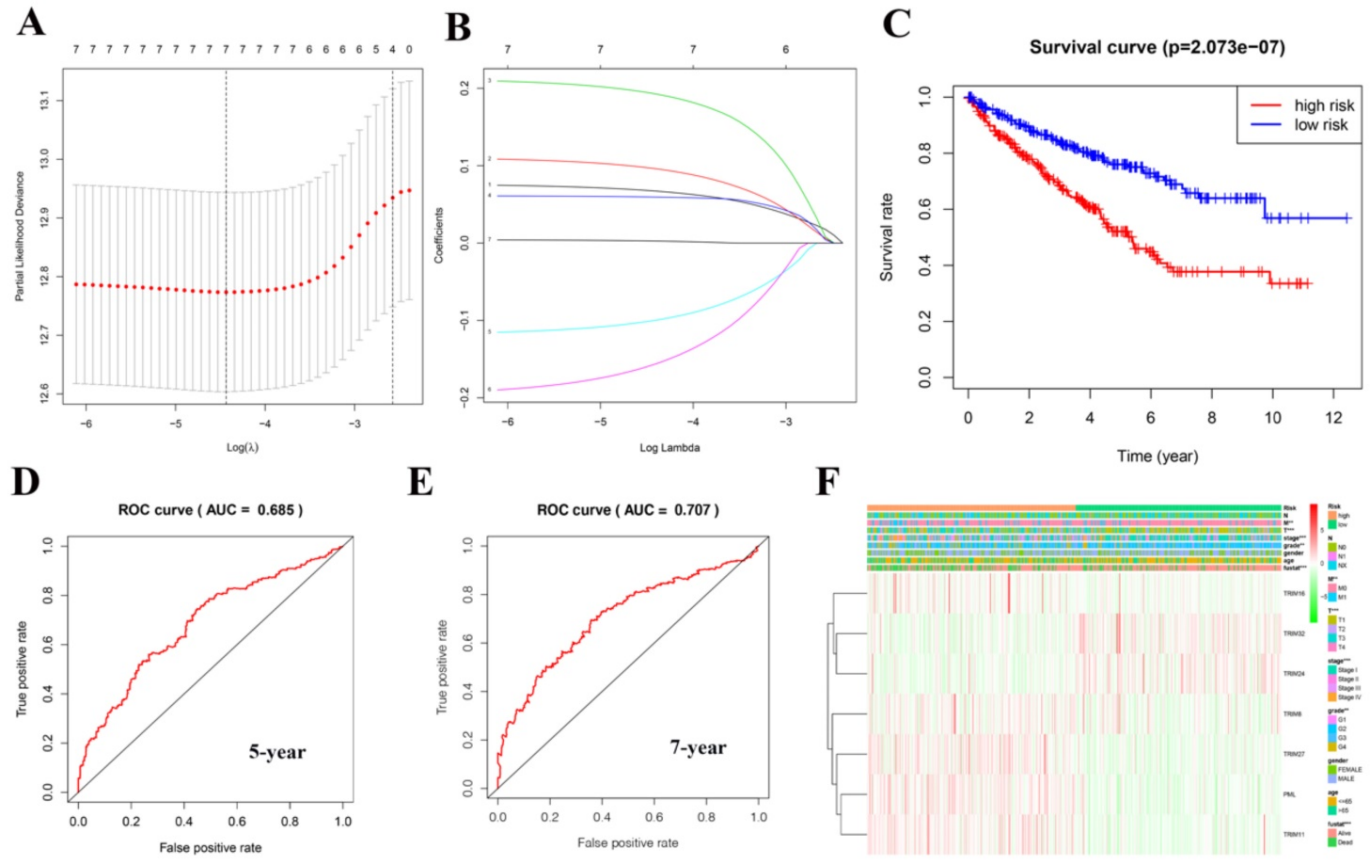
Creating a risk signature with seven target genes. (A, B) Lasso regression analysis curve and verification process. (C) The survival curve in patients with KIRC based on this risk model. (D) Five-year ROC curve. (E) Seven-year ROC curve. (F) The correlation with clinical characteristics based on this risk model. *P < 0.05, **P < 0.01, and ***P < 0.001.

A risk model was established using the Lasso regression analysis, and its feasibility was verified (Fig. 5A, 5B). The model consists of seven genes: TRIM16, TRIM32, TRIM24, TRIM8, TRIM27, PML, and TRIM11. Using this model and dividing the 539 patients with KIRC into high- and low-risk groups, we found that the high-risk group's prognosis was significantly worse than that of the low-risk group (P = 2.073e-07; Fig. 5C). Based on the results of the ROC curve, the better the AUC, the better the prediction performance of the model. For the five-year ROC curve of patients with KIRC, the AUC was 0.685 (Fig. 5D), and the seven-year ROC curve was equal to 0.707 (Fig. 5E), indicating that the model had high accuracy. Based on this risk model, we found that there was a correlation between it and metastasis (M), tumor (T), stage, grade, and fustat (Fig. 5F).

### The expression of seven target genes in renal cancer cell lines and Kaplan-Meier survival in KIRC

**Figure 6:**
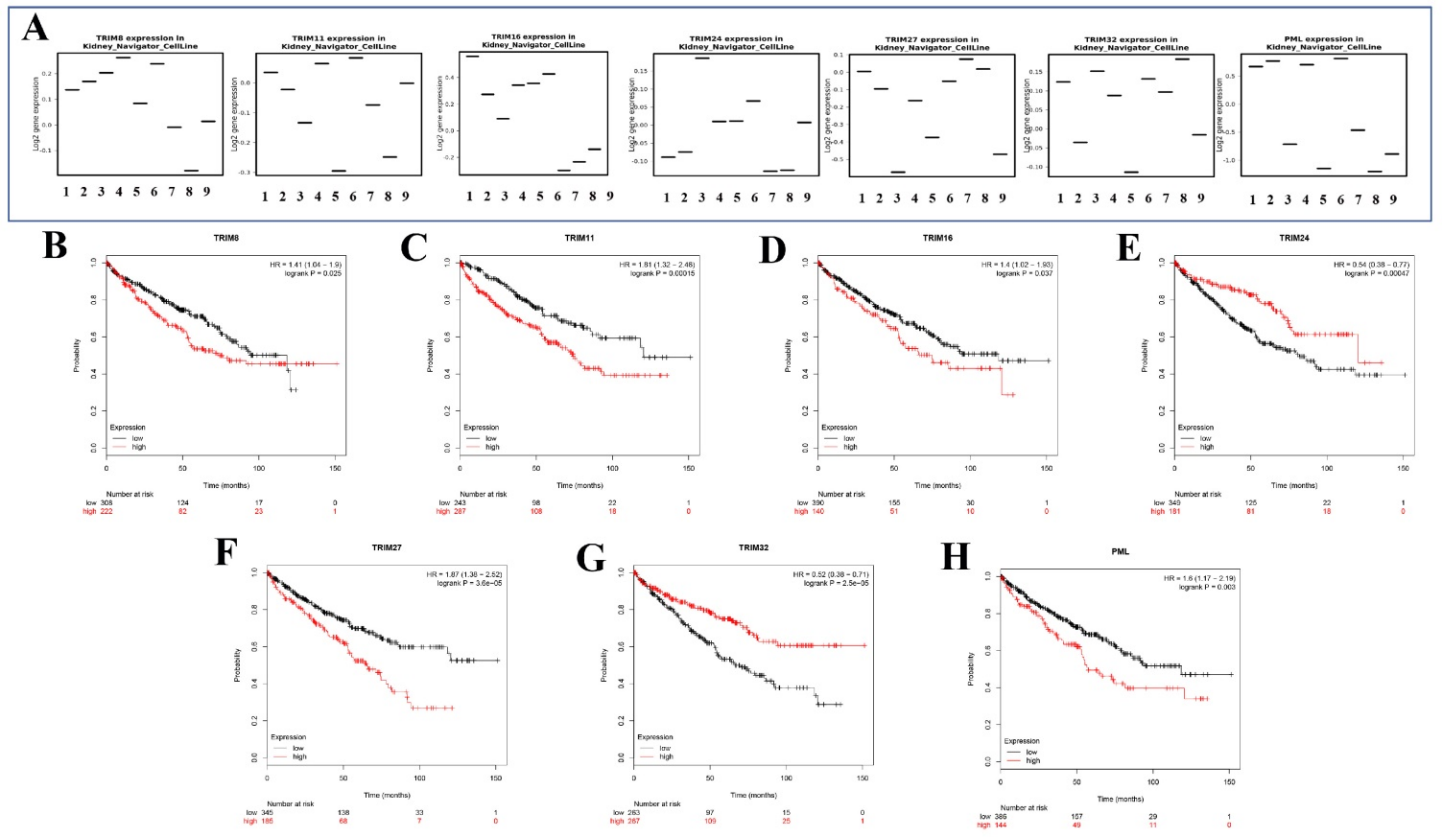
The expression in kidney cancer cell lines and the corresponding KM curve. (A) The expression of seven target genes (TRIM8, TRIM11, TRIM16, TRIM24, TRIM27, TRIM32, and PML) in various cell lines of renal cancer. (B-H) Kaplan-Meier curves of seven target genes (TRIM8, TRIM11, TRIM16, TRIM24, TRIM27, TRIM32, and PML) for patients with KIRC.

Seven genes (TRIM8, TRIM11, TRIM16, TRIM24, TRIM27, TRIM32, and PML) were expressed in different renal cancer cell lines through the KM expression website (Fig. 6A). The Kaplan-Meier survival curves of seven target genes in KIRC were obtained using the Kaplan-Meier Plotter website. These seven gene expressions were significantly associated with the prognosis of patients with KIRC (Fig. 6B). Among them, high TRIM8, TRIM11, TRIM16, TRIM27, and PML expression suggested that a patient had a poor prognosis. In contrast, high TRIM24 and TRIM32 expression indicated that the patient had a better prognosis.

### Drawing of a regulatory network diagram between target genes and transcription factors and establishment of a new nomogram based on the risk model in KIRC and random internal sampling validation

**Figure 7:**
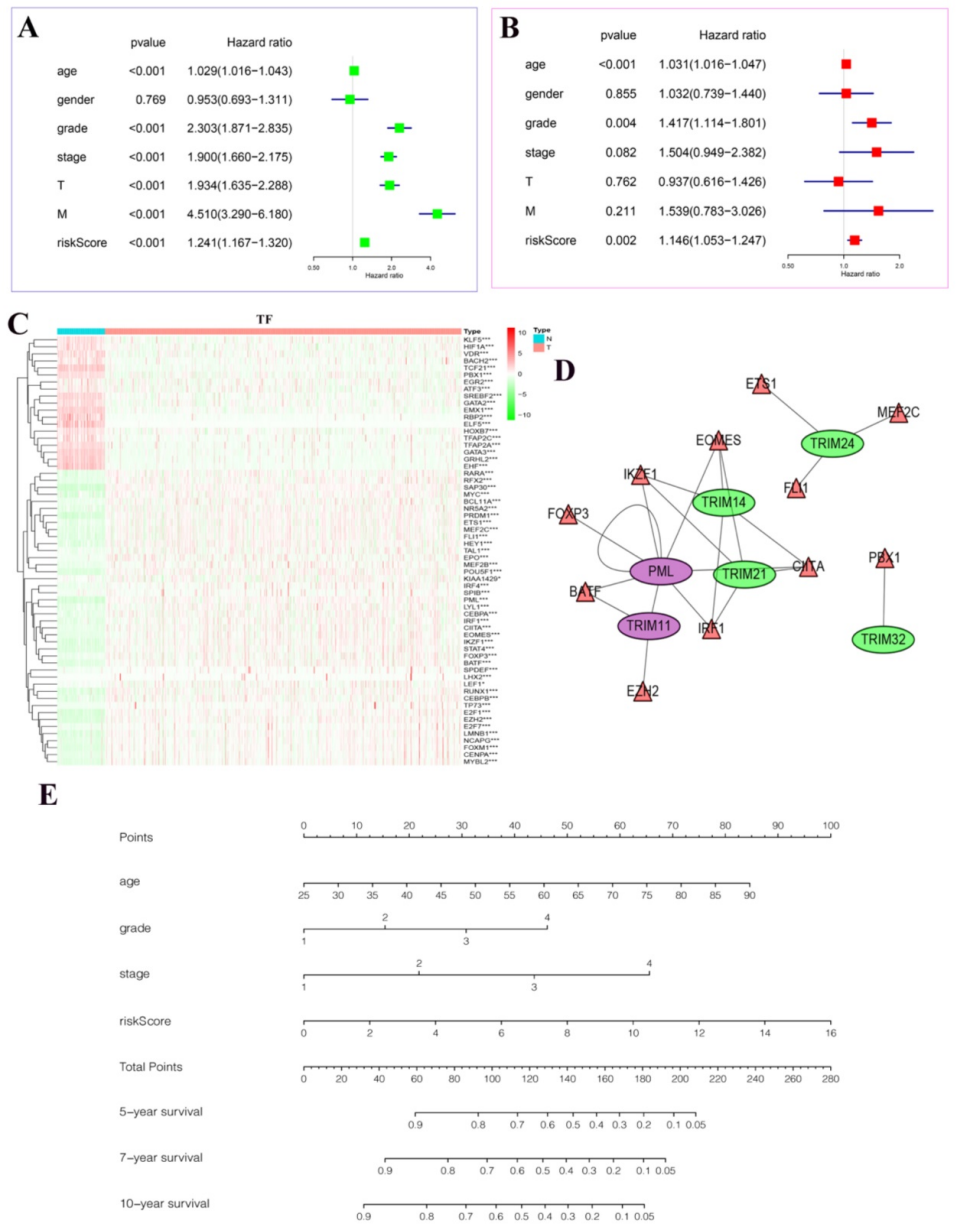
The results of Cox regression analyses, a regulatory network diagram between target genes and transcription factors, and a new nomogram based on the risk model in KIRC. (A) Univariate Cox regression analyses. (B) Multivariate Cox regression analyses. (C) The expression of transcription factors related to the seven target genes in KIRC. The deeper the shade of red, the higher the expression; the deeper the shade of green, the lower the expression. (D) A regulatory map between seven target genes and their related transcription factors. Purple represents activation and green represents inhibition. The triangle represents the transcription factor, and the ellipse represents the target gene. (E) A new nomogram was constructed based on this prognostic risk signature. The value of each variable received a score on the dot scale axis. The total score was easily calculated through the addition of each score and projecting the total score to a lower total score system. We estimated the risk for predicting five-, seven-, or 10-year survival in KIRC. *P < 0.05, **P < 0.01, and ***P < 0.001.

In univariate Cox regression analysis, we found statistical significance between age, grade, stage, T, M, riskScore, and patient prognosis (Fig. 7A). In multivariate Cox regression analysis, we found that age, grade, riskScore, and patient prognosis were statistically significant and were independent risk factors (Fig. 7B). In particular, we plotted heatmaps showing the expression of multiple transcription factors in patients with KIRC (Fig. 7C). Finally, the regulatory network between these transcription factors and target genes was plotted (Fig. 7D). Among them, we found that PML could be regulated not only by transcription factors but also by its regulation. The nomogram predicted the risk of patients with KIRC (Fig. 7E). The value of each variable presented a score on the dot-scale axis. The nomogram generated nine rows. The second, third, fourth, and fifth rows represent age, grade, stage, and risk score, respectively. The total score in the sixth row was obtained from the sum of each score assigned to age, grade, stage, and the risk score, and the five-, seven-, and 10-year survival rates of patients with KIRC were estimated from the total score. In addition, to increase the reliability of the results of this study, we conducted random internal sampling validation in the KIRC dataset of TCGA database. Based on this risk model, we divided the 218 randomly selected patients with KIRC into high- and low-risk groups. In the generated survival curve, we found that the prognosis of patients in the high-risk group was significantly worse than that of those in the low-risk group (P = 8.331e-05) (Fig. S1A). To verify the reliability of the model, we again performed ROC analysis. The results showed that the AUC value of the five-year ROC curve was 0.748, and the AUC value of the seven-year ROC curve was 0.796 (Fig. S1B-C). Correlation analysis of clinicopathological characteristics showed significant correlation between the model and the patient's T, stage, and fustat (Fig. S1D). Univariate Cox regression analysis and multivariate Cox regression analysis showed that the risk score of the model was a risk factor for the occurrence and development of KIRC (Fig. S1E-F). Therefore, the above series of results once again prove the reliability of the model.

## Discussion

Renal cell carcinoma (RCC) is one of the most common urological tumors worldwide. In 2019, there were approximately 73,820 new cases and approximately 14,770 deaths in the United States^23^. The status of RCC prevention and treatment in China is also not optimistic. The incidence and mortality of RCC in China are increasing. In 2015, there were approximately 66,800 new cases and 23,400 deaths in China relating to RCC^24^. According to the World Health Organization (WHO), the prognosis for RCC is poor, with approximately 90,000 deaths worldwide each year as a result^25^. KIRC is the primary form of adult kidney cancer and the most common subtype of RCC. Therefore, exploring the molecular mechanisms and new therapeutic targets of RCC remains an urgent issue.

At present, research on cancer stem cells (CSCs) is a hot topic in oncology. Cancer stemness refers to the presence of CSCs and an increased propensity to maintain CSC subpopulations, which is essential for tumors to build a resistance to treatment^26^, ^27^. Cancer stemming affects not only the lifespan of cancer cells but most importantly, enhances chemical resistance. The inherent strength of cancer cells may be due to the tendency of CSCs in having higher DNA repair abilities, cell quiescence, and promotion of drug efflux transporter expression. When CSCs survive an initial course of chemotherapy, they are likely to develop a chemical resistance phenotype. In addition, studies related to ubiquitination are currently popular in oncology. The TRIM family of proteins represents one of the largest classes of putative single protein RING-finger E3 ubiquitin ligases, which play an essential role in the ubiquitination of proteins. Many members of the TRIM family are cancer stemming.

In this study, we used a variety of bioinformatics tools to investigate the potential role of these TRIM family genes related to CSCs in pan-cancer and focus on KIRC. Here, we used the Lasso regression curve to establish a model associated with the prognosis of patients with KIRC that included seven genes; TRIM16, TRIM32, TRIM24, TRIM8, TRIM27, PML, and TRIM11. In addition, based on this risk model, we established a nomogram that can predict the five-, seven-, and 10-year survival rates of patients with KIRC. TRIM8, also known as RNF27 and GERP, is a highly conserved protein in the evolutionary process and is involved in a variety of biological processes, including carcinogenesis and inflammation^28^, and plays a vital role in innate immune pathway effects. In particular, TRIM8 regulates various signaling pathways through the direct interactions of key effectors, such as TAK1^29^, PIAS3^30^ and SOCS-1^31^. TRIM8 also acts as an oncogene in the regulation of the TNF-induced NF-κB pathway. Overexpression of TRIM11 promotes the growth and metastasis of lung cancer, glioma cells, hepatocellular carcinoma cells, osteosarcoma cells, and ovarian cancer cells^32^. After downregulating the expression of TRIM11 in breast cancer cells, improved therapeutic effects can be achieved by inhibiting the ERK1/2 and JNK1/2 signaling pathways^33^. TRIM11 directly interacts with miR-24-3p in colon cancer cells^34^. The most critical regulator in the ubiquitin-proteasome system appears to be TRIM16. Abnormal activation of the ubiquitin-proteasome system has been identified in many cancers^35^. Overexpression of TRIM16 can inhibit cell proliferation in most tumors, and this effect is often called tumor suppression. This protein's function is achieved by inhibiting the expression of E2F1 and pRb molecules^36^. Low expression of TRIM16 can promote squamous cell carcinoma of the head and neck^37^, lung cancer^38^. However, the high expression of TRIM16 is a marker of an adverse prognosis for ovarian cancer^39^. Through database analysis, we showed that the overall survival time of patients with KIRC in the TRIM16 high expression group was shorter than that in the low expression group. However, the underlying mechanism requires further exploration. The overexpression of TRIM27 can promote the malignant progression of colorectal cancer^40^. Moreover, the inhibition of TRIM27 expression can inhibit proliferation in ovarian, esophageal, and nasopharyngeal carcinoma cells^41^. In particular, TRIM27 positively regulates apoptosis induced by TNF-α^42^. PML, also known as TRIM19, is involved in the biological effects caused by IFN^43^ and interferes with the formation of HIV-1 complexes^44^.

In previous studies, the overexpression of TRIM24 promotes tumorigenesis and development in various tumors and it acts as a proliferation regulator in hepatocellular carcinoma and gastric cancer cells^45^, ^46^. The silencing of miR-137 increases the resistance of prostate cancer cells to androgen bicalutamide by directly upregulating TRIM24 expression^47^. However, in the present study, we found that patients with KIRC with low TRIM24 expression had shorter overall survival than those with high expression. The potential mechanism of TRIM24 in KIRC requires further exploration. TRIM32 plays an oncogene role in many cancers, including breast cancer, lung cancer, gastric cancer, and skin cancer^48^-^51^. Mechanistically, TRIM32 acts on tumor suppressors, such as P53 and Abi2, and regulates their degradation^52^, ^53^. In renal cancer, TRIM32 promotes cell proliferation and invasion by activating the β-catenin signaling pathway^49^. In neuroblastoma, TRIM32 interacts with MYCN and promotes its proteasomal degradation, which induces asymmetric cell division^54^. These seven target genes play various roles in the occurrence and development of multiple tumors. Therefore, they are expected to become targets for cancer treatment in future. However, the main limitation of our research is that my research is conducted at the level of bioinformatics. Further research is needed, especially in vitro and in vivo biological experiments, to verify the potential functions of these seven target genes in KIRC. In the future, we will conduct more in-depth research on these seven target genes in KIRC.

## Conclusions

In this study, we conducted an in-depth exploration of TRIM family genes related to CSCs and used seven TRIM family genes to build a model that can predict the prognosis of patients with KIRC. Based on this model, the AUC of the five-year ROC curve was found to be 0.685, and the AUC of the seven-year ROC curve was found to be 0.707, indicating that the model has an excellent predictive performance. Clinically, patients with KIRC can be divided into high- and low-risk groups according to this prognostic model. This will allow the development of a personalized treatment plan to prolong the survival time of the high-risk group. Patients in the high-risk group should be followed-up with more frequently, and relevant examinations should be regularly conducted to keep abreast of any changes in the disease.

## Supplementary Material

Supplementary figure.

## Abbreviations

TRIM: Tripartite-motif
KIRC: Kidney renal clear cell carcinoma
CNV: Copy number variation
TCGA: The Cancer Genome Atlas
SNV: Single nucleotide variation
GO: Gene ontology
KEGG: Kyoto encyclopedia of genes and genomes
BP: Biological process
CC: Cellular component
MF: Molecular function
CSCs: Cancer stem cells
RCC: Renal cell carcinoma
TRIM6: Tripartite Motif Containing 6
TRIM8: Tripartite Motif Containing 8
TRIM11: Tripartite Motif Containing 11
TRIM14: Tripartite Motif Containing 14
TRIM16: Tripartite Motif Containing 16
PML: PML Nuclear Body Scaffold
TRIM21: Tripartite Motif Containing 21
TRIM24: Tripartite Motif Containing 24
TRIM25: Tripartite Motif Containing 25
TRIM27: Tripartite Motif Containing 27
TRIM28: Tripartite Motif Containing 28
TRIM32: Tripartite Motif Containing 32
TRIM71: Tripartite Motif Containing 71
