# Supplementary material for: Tripartite-motif family genes associated with cancer stem cells affect tumor progression and can assist in the clinical prognosis of kidney renal clear cell carcinoma
Source: Int J Med Sci. 2020 Oct 18;17(18):2905–16. doi: 10.7150/ijms.51260 (PMC7646106; doi:10.7150/ijms.51260)
Supplement: Supplementary file 1 — Supplementary figure. [file ijmsv17p2905s1.pdf]

Supplementary materials

Figure S1: Random sampling validation. (A) Based on this risk model, 218 patients with KIRC randomly sampled from TCGA database were divided into high- and low-risk groups, and the corresponding survival curves were drawn. (B) Five-year ROC curve. (C) Seven-year ROC curve. (D) Heatmap. The previous correlation analysis with clinicopathological characteristics based on the risk model. (E) Univariate Cox analysis. (F) Multivariate Cox analysis. *$P < 0.05$ and ***$P < 0.001$.

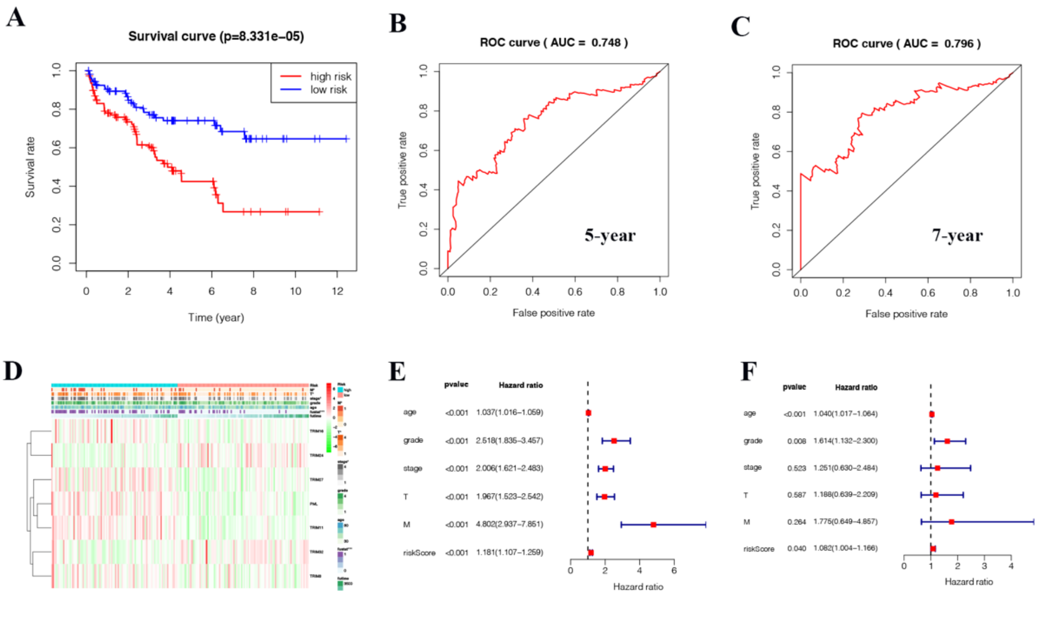
A
Survival curve (p=8.331e−05)
high risk
low risk
Survival rate
Time (year)
B
ROC curve (AUC = 0.748)
True positive rate
False positive rate
5-year
C
ROC curve (AUC = 0.796)
True positive rate
False positive rate
7-year
D
E
pvalue
Hazard ratio
age <0.001 1.037(1.016−1.059)
grade <0.001 2.518(1.835−3.457)
stage <0.001 2.006(1.621−2.483)
T <0.001 1.967(1.523−2.542)
M <0.001 4.802(2.937−7.851)
riskScore <0.001 1.181(1.107−1.259)
Hazard ratio
F
pvalue
Hazard ratio
age <0.001 1.040(1.017−1.064)
grade 0.008 1.614(1.132−2.300)
stage 0.523 1.251(0.630−2.484)
T 0.587 1.188(0.639−2.209)
M 0.264 1.775(0.649−4.857)
riskScore 0.040 1.082(1.004−1.166)
Hazard ratio

Figure S1
